# Light-Induced Changes in Fatty Acid Profiles of Specific Lipid Classes in Several Freshwater Phytoplankton Species

**DOI:** 10.3389/fpls.2016.00264

**Published:** 2016-03-16

**Authors:** Alexander Wacker, Maike Piepho, John L. Harwood, Irina A. Guschina, Michael T. Arts

**Affiliations:** ^1^Theoretical Aquatic Ecology and Ecophysiology, Institute of Biochemistry and Biology, University of PotsdamPotsdam, Germany; ^2^Department for Ecology, Institute of Aquatic Ecology, University of RostockRostock, Germany; ^3^School of Biosciences, Cardiff UniversityCardiff, UK; ^4^Department of Chemistry and Biology, Ryerson UniversityToronto, ON, Canada

**Keywords:** freshwater algae, light adaptation, lipid classes, fatty acid changes

## Abstract

We tested the influence of two light intensities [40 and 300 μmol PAR / (m^2^s)] on the fatty acid composition of three distinct lipid classes in four freshwater phytoplankton species. We chose species of different taxonomic classes in order to detect potentially similar reaction characteristics that might also be present in natural phytoplankton communities. From samples of the bacillariophyte *Asterionella formosa*, the chrysophyte *Chromulina* sp., the cryptophyte *Cryptomonas ovata* and the zygnematophyte *Cosmarium botrytis* we first separated glycolipids (monogalactosyldiacylglycerol, digalactosyldiacylglycerol, and sulfoquinovosyldiacylglycerol), phospholipids (phosphatidylcholine, phosphatidylethanolamine, phosphatidylglycerol, phosphatidylinositol, and phosphatidylserine) as well as non-polar lipids (triacylglycerols), before analyzing the fatty acid composition of each lipid class. High variation in the fatty acid composition existed among different species. Individual fatty acid compositions differed in their reaction to changing light intensities in the four species. Although no generalizations could be made for species across taxonomic classes, individual species showed clear but small responses in their ecologically-relevant omega-3 and omega-6 polyunsaturated fatty acids (PUFA) in terms of proportions and of per tissue carbon quotas. Knowledge on how lipids like fatty acids change with environmental or culture conditions is of great interest in ecological food web studies, aquaculture, and biotechnology, since algal lipids are the most important sources of omega-3 long-chain PUFA for aquatic and terrestrial consumers, including humans.

## Introduction

Fatty acids (FA) are major components of most of the important lipids in eukaryotic organisms. There are two major divisions of lipids: non-polar lipids and polar lipids. Non-polar lipids (NL) for the most part consist of triacylglycerols and wax esters; the main energy storage molecules of aquatic organisms. The most important polar lipids of phytoplankton are structural components of cell membranes and include, phospholipids (PL), glycolipids (GL), and betaine lipids. PL are predominantly found in non-chloroplast membranes (plasma membrane, endoplasmic reticulum, etc.), whereas GL are typical constituents of chloroplast thylakoid membranes (Guschina and Harwood, 2009). Betaine lipids are common in algae and lower plants and have been observed in plasma and chloroplast membranes (Sato, 1992, Haigh et al., 1996). Not much is known about their function, but, like PL, they seem to be structural components of membranes and it has been tentatively suggested that, unlike PL, they might not be affected by light intensity (Khotimchenko and Yakovleva, 2004). Apart from their structural role, FA and their associated lipids are also important functional molecules. For example, they are involved in signal transduction (Sumida et al., 1993) and in photosynthesis (Harwood and Jones, 1989; Sukenik et al., 1989; Jordan et al., 2001; Dörmann and Benning, 2002; Jones, 2007). FA composition of the different lipid classes varies with species and with environmental conditions. These variations in biochemical composition allow algae to survive as environmental conditions change.

It has been shown that the lipid structure of thylakoid membranes plays a central role for the organization of the light harvesting complex and the photosynthetic performance of algae (Pronina et al., 1998; Goss and Wilhelm, 2009; Schaller et al., 2011). In addition to the lipid class itself, the FA composition within each lipid class is also important (Li et al., 2012); polyunsaturated fatty acids (PUFA) are important in adjusting membrane fluidity and with it the electron flow between electron acceptors of photosystem II (Horváth et al., 1987; Mock and Kroon, 2002; Guiheneuf et al., 2009). In photosynthetically active membranes, there are two compositional responses (with respect to FA) to light that act in opposite directions. Under low light acclimation higher proportions of PUFA reflect an increase in thylakoid membrane stacking (see e.g., Richardson et al., 1983; Neale and Melis, 1986; Sakshaug et al., 1997), whereas lower proportions of PUFA are associated with a decrease of proton leakage to reduce metabolic costs (Raven et al., 2000; Quigg et al., 2006).

Elucidating the general mechanisms underlying the biochemical adaptations made by algae in response to changing environmental conditions is important in order that we might be in a better position to predict variation in algal food quantity and quality in aquatic food webs experiencing extensive impacts of anthropogenic eutrophication (Schindler, 2012) and climate change (Fuschino et al., 2011). This is particularly important in estimating potential effects of these global drivers on biodiversity because different phytoplankton species occupy distinct physico-chemical niches allowing for higher resource use efficiency (Striebel et al., 2009). Thereby species-specific responses of phytoplankton in response to fluctuations in their immediate environment have to be quantified and characterized. For example, representatives of the Chlorophyceae, Cryptophyceae, and Mediophyceae, all of which are important components of plankton communities in temperate lakes, showed different photosynthetic acclimation strategies to changing abiotic environmental conditions such as phosphorus availability and light intensity (Wacker et al., 2015a). Second, phytoplankton species have been shown to be highly variable with respect to their FA composition and sterols when nutrient concentration, light intensity and temperature fluctuate (Piepho et al., 2010, 2012a,b). Consequently, changing environmental conditions may cause alterations in relative availabilities of mineral and biochemicals that are essential for higher trophic levels (Striebel et al., 2012; Wacker et al., 2015b).

Increased nutrient input (eutrophication) and increasing temperature (climate change) favor cyanobacterial blooms that may significantly reduce the amount of light penetrating into the water (Paerl and Huisman, 2008). Effects of climate change on water clarity, however, can have other underlying causes. For example, decreasing precipitation could reduce input of organic matter into lakes and thus increase water clarity (Gunn et al., 2001). In either case phytoplankton cells may react by changing their behavior, adjusting their light optima and growth rates (Clegg et al., 2003). Interspecific interactions between different phytoplankton species may further influence their light acclimation and will also change their biochemical composition. This is indicated by a recent study showing that, under competitive stress, algae produced either lower or higher amounts of specific fatty acids depending on the species examined (Wacker et al., 2015b). Although the mechanistic drivers actually at play still have to be identified, we expect that changes in incident light and nutrient availability affected the concentration and composition of pigments (Richardson et al., 1983; Striebel et al., 2009), the photosynthetic efficiency (Wacker et al., 2015a), as well as the cellular FA and sterol composition (Piepho et al., 2010, 2012a,b). To date, it has been difficult to generalize effects of environmental conditions on algal FA, mostly due to their highly species-specific FA profiles; additionally, reactions to changing conditions have been found to be highly variable (Piepho et al., 2012a). For example, alterations in PUFA concentrations to light limitation have been contradictory in several studies performed with different species: some increase their PUFA concentration under light limitation (Sukenik et al., 1989; Mock and Kroon, 2002; Guiheneuf et al., 2009), while others increase PUFA concentrations in high light (Seto et al., 1984; Zhukova, 2007; Solovchenko et al., 2008). Such species-specific adjustments in FA concentrations in response to changing environmental conditions might be based on a species-specific light acclimation strategy reflected in photosynthetic parameters and differences in light optima (Falkowski and Owens, 1980; Richardson et al., 1983; Gervais, 1998; Raven et al., 2000; Clegg et al., 2003; Wacker et al., 2015a). Additionally, a general, across species, pattern of FA acclimatization to light might have been masked by the fact that in many studies FA composition is often related only to bulk lipids. As FAs are integral components of many different lipid classes, that differ in their overall function, acclimatization to varying environmental conditions might occur at the level of lipid classes. The n-3 PUFA are main components of the thylakoid membrane-forming galactolipids such as monogalactosyldiacylglycerol (MGDG) and digalactosyldiacylglycerol (DGDG). If species are using the prokaryotic synthesis pathway then both 16- and 18-carbon fatty acids (e.g., 16:3n-3 and 18:3n-3) are most frequently esterified to the sn-1 and sn-2 positions of the thylakoid galactolipids, respectively. If, however, organisms use the eukaryotic pathway, then galactolipids will contain the 18-carbon fatty acid in both the sn-2 and sn-1 positions (Harwood and Jones, 1989; Harwood, 2004; Goss and Wilhelm, 2009). At low light phytoplankton may optimize their photosynthesis (Falkowski and Owens, 1980), and such active optimization is coupled with a rise in n-3 desaturation of FAs and an increase of e.g., the above-mentioned n-3 PUFA (Klyachko-Gurvich et al., 2000). Consequently, we expect an increase of 16:3n-3 and/or 18:3n-3 in galactolipids under optimization to low-light. Additionally, the sulfolipid sulfoquinovosyldiacylglycerol (SQDG) and the phospholipids phosphatidylcholine (PC), phosphatidylethanolamine (PE), phosphatidylglycerol (PG), phosphatidylinositol (PI), and phosphatidylserine (PS) may be affected.

In this study we analyzed the lipid class-specific FA compositions of four freshwater phytoplankton species cultured under two different light intensities. Our aim was to determine if we could discern, under rigorously standardized conditions, common patterns in the responses of FAs, in specific lipid classes, to light in phytoplankton species originating from different taxonomic classes. We tested phytoplankton species of four different taxonomic classes, including classes that have rarely been represented in the literature before (e.g., Chrysophyceae and Zygnematophyceae). Although rarely studied, knowledge on species that are gaining in importance in the context of global climate change (Suikkanen et al., 2013) is essential. An explicit recognition of common patterns would increase our understanding of the general adjustments in the lipid and FA composition that algae would be predicted to make with changing light conditions. Our data emphasize the high variability of physiological responses and contribute to our understanding of adaptation in these vital photosynthetic eukaryotes.

## Materials and methods

### Cultures

The FA composition of different lipid classes in four freshwater phytoplankton species was analyzed after growth at two different light intensities. We chose algal species that are common in temperate freshwater lakes including: *Asterionella formosa* Hassall (Bacillariophyceae) (collection of algal cultures, Göttingen, Germany, SAG 8.95), *Chromulina* sp. (Chrysophyceae) (SAG 17.97), *Cryptomonas ovata* Ehrenberg (Cryptophyceae) (SAG 979-3), and *Cosmarium botrytis* Meneghini (Zygnematophyceae) (SAG 136.80). The species were cultivated in 1 L Erlenmeyer flasks each in 500 ml WC-medium (Nichols, 1973) in semi-continuous cultures, i.e. we diluted the cultures every day at the same time. Dilution rate was 0.2 d^−1^ for *Chromulina* sp. and *C. ovata*, 0.1 d^−1^ for *C. botrytis*, and 0.05 d^−1^ for *A. formosa*. These differences in dilution were necessary because of species-specific differences in growth rates. For *A. formosa* the medium was enriched with Si (200 μM Si instead of 100 μM Si in the other media; Si was provided in the form of Na_2_SiO_3_). Since this resulted in a change in pH, we re-adjusted to pH 7 by adding 1M HCl. A climate chamber (Vötsch GmbH, Balingen-Frommern, Germany, VB 1514; fluorescent lamps: Osram FLUORA L30W/77 and Osram LUMILUX L30W/830, warm white, Osram, Munich, Germany) was divided into two compartments with light intensities of 40 and 300 μmol PAR photons m^−2^ s^−1^, respectively (datalogger: LI-COR Environmental GmbH, Bad Homburg, Germany, LI-1400, equipped with a 4π quantum sensor) using neutral density foil filters (Lee Filters, Hampshire, England). Three replicates of each alga were grown in both compartments at 20°C. Although light intensities in lakes can reach much higher values than applied here, total lipid synthesis in algae has been reported to reach a threshold at light intensities ranging from 300 to 800 μmol photons m^−2^ s^−1^ (Wainman et al., 1999). Therefore, we chose conditions that should encompass one light-limited and one light-saturated treatment with respect to total lipid synthesis. In order to avoid daily fluctuations and short term adaptations in the biochemical composition of algal cells we applied continuous lighting, although we are aware that such conditions are not natural. Imposing a diurnal light cycle would have entailed synchronization of cell division rates (circadian rhythm) and with it great daily fluctuations in biochemical composition dependent on the stage of the cell cycle. Continuous light conditions desynchronize algal cell division patterns (Roenneberg et al., 1989) and therefore lead to an average biochemical composition that is constant over time. Optical density (OD 800 nm; UV mini-1240, Shimadzu, Duisburg, Germany) was measured daily and the growth rate was calculated (μ = [ln(OD_2_) – ln(OD_1_)]/t; μ: growth rate, OD_1_: OD (800 nm), OD_2_: OD (800 nm) after time t). All experiments were carried out until growth rates of cultures remained constant (17–24 days) to ensure that all cells of a culture had the same cell division rate and to allow the cells to acclimate to experimental conditions. Carbon deficiency was avoided by aerating the media with sterile filtered-air.

### POC determination

Particulate organic carbon (POC) was measured by first filtering ~0.25 μg carbon of the algal suspension onto 25 mm, pre-combusted, glass fiber filters (Whatman, GF/F, Dassel, Germany) and then quantifying algal C using an elemental analyzer (HEKAtech GmbH, Wegberg, Germany, Euro EA 3000).

### Separation and identification of lipids

Separation and identification of lipids was done accordingly to Hahn-Deinstrop (2006). Lipid samples were obtained by filtering ≥3 mg algal carbon onto glass fiber filters (Whatman, GF/F). Filters were stored at −25°C under nitrogen atmosphere in glass tubes with Teflon seals after adding 7 ml of dichloromethane-methanol (2:1 v/v) for extraction of lipids. Lipid extracts were concentrated prior to separation of lipid classes. The dichloromethane-methanol solution was evaporated by placing the tubes in a metal block thermostatically controlled at 40°C (Kleinfeld Labortechnik, Gehrden, Germany, MBT-100) and with simultaneous aeration with nitrogen gas to avoid reaction of lipids with atmospheric oxygen. Lipid samples were re-suspended in 30 μl chloroform and stored ≤1 day at −25°C. Separation of PL, GL and NL was achieved by two-dimensional thin-layer chromatography (TLC). Samples were applied to 20 × 20 cm aluminum silica gel plates (Merck KGaA Darmstadt,Germany, 105553) and separated using chloroform-methanol-water (65:25:4, v/v/v) in the first dimension and chloroform-methanol-acetic acid-water (80:9:12:2, v/v/v/v) in the second dimension. For identification of lipid classes, standards of DGDG (Sigma, D4651, Sigma-Aldrich Chemie, Taufkirchen, Germany), L-α-PC (Sigma, P3556), L-α-PI (Sigma, P5766), L-α-PE (Sigma, P7943), L-α-phosphatidyl-DL-glycerol (Sigma, P8318), and triacylglycerol (Sigma, 17811) were also applied to the TLC plates.

Replicate lipid samples were used to identify lipid classes by various spray reagents: ninhydrin reagent (Carl Roth GmbH + Co. KG, Karlsruhe, Germany, CP30.1) for visualization of lipids containing amino acids, such as PE and PS, Dragendorff-Munier reagent (Roth GmbH + Co. KG, CP33.1) for lipids containing tertiary nitrogen compounds, such as PC, and orcinol reagent in 2M H_2_SO_4_ (Sigma, 447420) for identification of free carbohydrates and glycoconjugates, such as in MGDG, DGDG, and SQDG. Organically bound phosphorus, thus including PL, was additionally visualized using a molybdenum blue spray reagent (Sigma, M1942). We identified each lipid class repeatedly by the described methods to be sure about the results. In addition to authentic standards and spray reagents we calculated retention factors (R_F_-values) and, where possible, compared them with literature data. As it was suggested that betaine lipids might be unaffected by light intensity (Khotimchenko and Yakovleva, 2004), we did not further analyze betaine lipids.

A parallel set of TLC plates, destined to be used for quantification of FA, were lightly stained using iodine gas, in which unsaturated lipids appeared as brown spots. This reaction is, in contrast to the above described spray reagents, reversible and does not destroy lipid molecules (Barrett, 1962; Kates, 1986) making it a good method for subsequent analyses by gas chromatography. The different lipid spots were scraped off the plates and transferred into glass tubes with Teflon seal under nitrogen atmosphere after adding 7 ml of dichloromethane-methanol (2:1 v/v).

### Fatty acid analyses

From samples of *A. formosa, Chromulina* sp., *C. ovata*, and *C. botrytis* we first separated glycolipids (MGDG, DGDG, and SQDG), phospholipids (PC, PE, PG, PI, and PS) as well as non-polar lipids (triacylglycerols), before analyzing the fatty acid composition of each lipid class.

FA were analyzed separately for the total FA fraction as well as for the TLC-separated non-polar lipids (triacylglycerols), glycolipids (MGDG, DGDG, and SQDG), and phospholipids (PC, PE, PG, PI, and PS). Lipid extraction was done twice with dichloromethane-methanol (2:1 v/v, c.f. Cequier-Sánchez et al., 2008). No heat treatment or salt washing step was used since we could not find differences in the efficiency of lipid extraction between these methods and ours (preliminary experiment, data not shown). Transesterification was done according to a modified version of the procedure described by Mason and Waller (1964) by adding 4 ml methanolic HCl to the dried lipid sample and heating it under nitrogen gas (20 min, 60°C). Identification and quantification of FA methyl esters (FAME) was done by gas chromatography (Agilent Technologies, Böblingen, Germany, 6890N) according to Wacker and Weithoff (2009) but with the following configuration: 1 μl of sample was injected in split mode (5:1), vaporized in the injector at 250°C and mixed with the carrier gas (helium). FAME were separated on a 50% cyanopropyl-phenyl methyl-polysiloxane column (Agilent Technologies J&W DB-225, 30 m × 0.25 mm × 0.25 μm) using the following temperature gradient; 60°C for 1 min, increasing at 20°C min^−1^ until 150°C, 10°C min^−1^ until 220°C and then held for 13.75 min. FAME were detected using a flame ionization detector (FID) at 250°C. FAME were quantified using multipoint standard calibration curves determined for each FAME (using six different concentrations per FAME) from mixtures of known composition (Supelco® 37 Component FAME Mix, 47885-U, Sigma-Aldrich Chemie). Identification of FAME from the samples was done routinely via known retention times of the Supelco® FAME Mix reference substances. We also used mass spectra for confirmation, which were recorded with a gas chromatograph-mass spectrometer (Thermo Scientific, Dreieich, Germany, Finnigan MAT GCQ) equipped with a fused-silica capillary column (Agilent Technologies J&W DB-225 ms; see Martin-Creuzburg et al., 2009).

### Statistics

FA concentrations of individual lipid classes were expressed in percent of the sum of FA within each lipid class. Principal component analysis (PCA) was used for each phytoplankton species separately to identify potential differences between fatty acid compositions of lipid classes. Saturated and unsaturated fatty acids that were detected in significant amounts in the lipid classes of the majority of the studied species were 16:0, 16:1n-9, 16:3n-3, 18:0, 18:1n-7, 18:1n-9, 18:2n-6, 18:3n-6, 18:3n-3, 18:4n-3, 20:4n-6, and 20:5n-3. These were scaled and included as variables in the PCA. Analysis of variance (ANOVA) was used to test for significant differences of (arc-sin-transformed) fatty acid proportions between the two light conditions. All statistical calculations were carried out using the software package R (R Core Team, 2013, version 3.02).

## Results

The fatty acid composition in total lipids of *A. formosa, Chromulina* sp., *C. botrytis*, and *C. ovata* was highly species-specific (Table 1, Supplementary Figure 1, Supplementary Table 1). *A. formosa* contained high proportions of 16:1n-9 and the long-chain PUFA 20:5n-3 and did not show any pronounced adjustments of the FA proportions to low light conditions. *Chromulina* sp. had high proportions of 18-carbon PUFAs (i.e., 18:2n-6, 18:3n-6 and 18:3n-3, and 18:4n-3) and of the long-chain PUFA 20:4n-6 (Table 1). The proportion of 18:3n-3 was ~two-fold higher under low light, while the proportions of 20:5n-3 and 22:6n-3 decreased ~2–3 fold. *C. botrytis* showed high proportions of 16:3n-3, 18:1n-9, and 18:3n-3, and did not change with light conditions. Distinct increases of fatty acid proportions under low light conditions were observed in 18:1n-9, which almost doubled. In *C. ovata* the (already high) proportions of the PUFA 18:4n-3 and the long-chain PUFAs 20:5n-3 and 22:6n-3 increased under low light conditions; the PUFA 18:3n-3 stayed constant.

**Table 1:** **Fatty acid proportions (%) in total fatty acids of the four analyzed phytoplankton species grown under different light acclimation (40 and 300 μmol photons m^**−2**^ s^**−1**^)**.

**Species**	***A. formosa***		***Chromulina*** **sp**.		***C. botrytis***		***C. ovata***	
**Light**	**40**	**300**	**40**	**300**	**40**	**300**	**40**	**300**
16:0	19.7 (±2.5)	21.0 (±0.6)	9.7 (±0.4)	10.3 (±0.3)	26.2 (±0.2)	27.2 (±0.9)	22.6 (±1.1)	22.9 (±0.7)
16:1n-9	**44.6 (±1.4)**	**38.0 (±3.1)**	**2.1 (±0.1)**	**3.1 (±0.4)**	**0.7 (±0)**	**1.3 (±0)**	**1.2 (±0.2)**	**2.6 (±0.1)**
16:3n-3	2.4 (±0.3)	1.8 (±0.3)	0.7 (±0)	0.9 (±0.1)	16.1 (±0.6)	15.3 (±0.9)	0.8 (±0.1)	0.9 (±0.1)
16:4n-3	**0.6 (±0)**	**0.8 (±0.1)**	**0.7 (±0)**	**1.2 (±0.1)**	0.4 (±0.1)	0.3 (±0)	**0.8 (±0)**	**1.2 (±0.1)**
18:0	2.9 (±0.9)	3.2 (±2.1)	1.9 (±0.1)	2.1 (±0.3)	1.3 (±0.2)	1.2 (±0.1)	2.9 (±0.6)	4.1 (±1.1)
18:1n-7	2.9 (±0.3)	3.3 (±0.6)	**2.3 (±0)**	**3.2 (±0.1)**	0.7 (±0)	0.8 (±0)	**1.9 (±0.3)**	**2.7 (±0.2)**
18:1n-9	2.9 (±0.1)	3.3 (±0.4)	**1.5 (±0)**	**2.0 (±0.3)**	**11.2 (±2.1)**	**6.9 (±0.8)**	**1.6 (±0.2)**	**3.2 (±0.4)**
18:2n-6	2.1 (±0.6)	2.2 (±0.2)	**7.2 (±0.4)**	**5.4 (±0.4)**	**5.7 (±0.2)**	**6.2 (±0.1)**	**3.0 (±0.4)**	**4.1 (±0.3)**
18:3n-6	1.4 (±0.3)	1.6 (±0.1)	**10.6 (±1.2)**	**5.1 (±0.5)**	3.1 (±0.1)	3.1 (±0.2)	n.d.	n.d.
18:3n-3	1.1 (±0.1)	1.2 (±0.2)	**10.4 (±0.4)**	**6.3 (±0.6)**	**23.3 (±0.5)**	**24.6 (±0.4)**	23.9 (±0.5)	26.0 (±1.8)
18:4n-3	1.0 (±0.2)	1.1 (±0)	35.5 (±0.7)	36.8 (±1)	**3.8 (±0.4)**	**5.3 (±0.6)**	**21.3 (±0.5)**	**16.9 (±1.0)**
20:0	**1.2 (±0.1)**	**1.6 (±0.1)**	**1.6 (±0)**	**2.1 (±0.3)**	n.d.	n.d.	n.d.	n.d.
20:1n-9	n.d.	n.d.	n.d.	n.d.	n.d.	n.d.	1.4 (±0.1)	1.4 (±0.1)
20:3n-6	0.9 (±0.5)	1.1 (±0.4)	**1.1 (±0)**	**1.4 (±0.1)**	n.d.	n.d.	0.9 (±0.5)	1.3 (±0.6)
20:4n-6	2.3 (±0.4)	2.8 (±0.3)	10.7 (±0.4)	9.7 (±0.6)	**1.1 (±0.1)**	**0.8 (±0)**	n.d.	n.d.
20:5n-3	10.0 (±3.0)	11.8 (±0.2)	**2.6 (±0.1)**	**6.1 (±0.7)**	5.7 (±0.9)	6.1 (±0.4)	**13.6 (±0.3)**	**10.0 (±0.8)**
22:0	1.0 (±0.1)	1.3 (±0.2)	n.d.	n.d.	0.7 (±0.1)	0.7 (±0.1)	n.d.	n.d.
22:6n-3	1.4 (±0.2)	1.8 (±0.3)	**1.5 (±0.1)**	**4.5 (±0.2)**	n.d.	n.d.	**4.1 (±0.7)**	**2.6 (±0.4)**
24:0	1.7 (±0.2)	2.2 (±0.3)	n.d.	n.d.	n.d.	n.d.	n.d.	n.d.

*Data shown are means and standard deviations (n = 3). Numbers in bold/red font indicate statistically significant differences of fatty acid proportions between the two light conditions (ANOVA, p < 0.05). Numbers in bold/blue font indicate statistically significant differences after sequential Bonferroni correction with an effective alpha of 5%. n.d. = not detected*.

**Figure 1:**
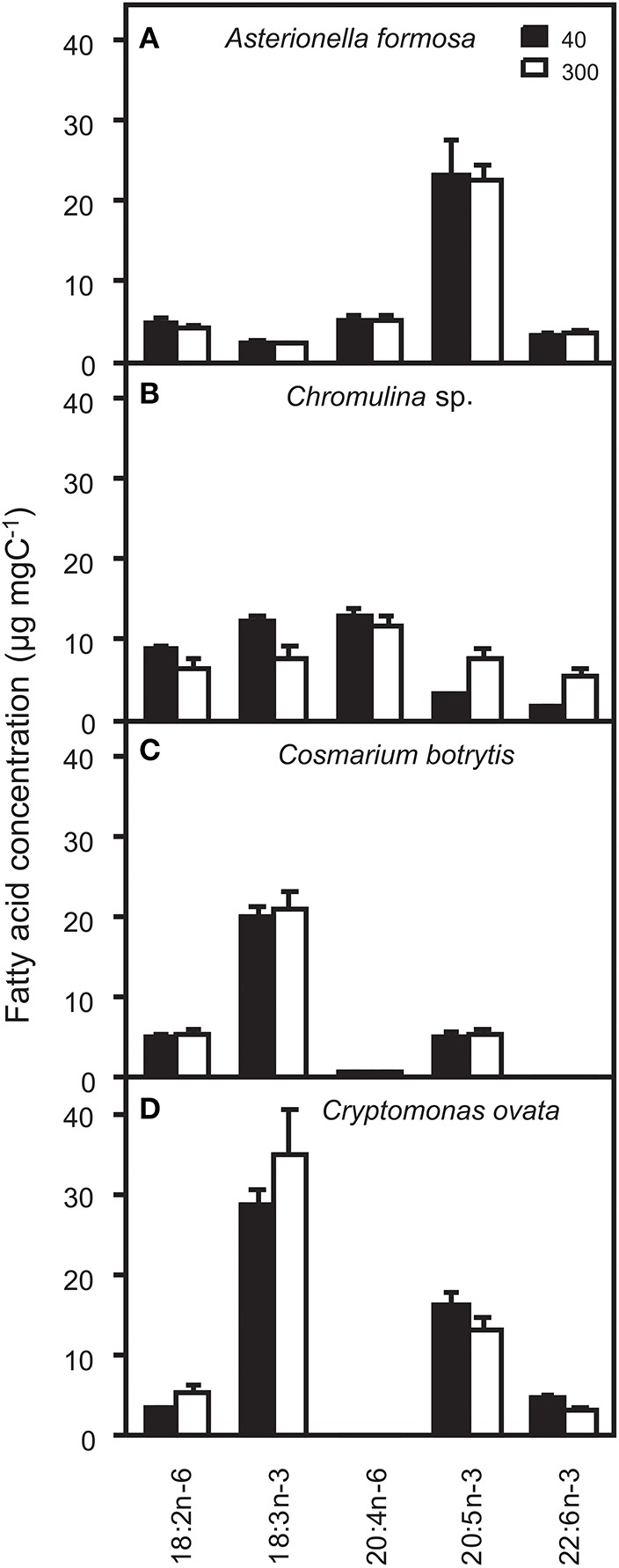
**Concentrations of ecologically relevant n-3 and n-6 polyunsaturated fatty acids (PUFA) on a per carbon basis (μg _**fatty acid**_ mg _**algal**_ C^**−1**^) and analyzed from the total lipid fractions of (A) ***Asterionella formosa***, (B) ***Chromulina*** sp., (C) ***Cosmarium botrytis***, and (D) ***Cryptomonas ovata*** cultured at 40 and 300 μmol photons m^**−2**^ s^**−1**^**.

The potential ecological impacts of changes in, for zooplankton relevant, n-3 and n-6 fatty acids were estimated by additionally calculating the fatty acid concentrations on a per tissue carbon basis. Depending on the for zooplankton grazers potentially limiting PUFA the light acclimation of the algae may have positive or negative effects on zooplankton growth. Such effects may not be induced by *A. formosa* and *C. botrytis* because per carbon concentration changes were negligible (Figure 1). This was different for *Chromulina* sp., which showed pronounced responses; for animals generally essential PUFAs 18:2n-6 and 18:3n-3 increased under low light conditions. Simultaneously, the two ecologically important and potentially zooplankton growth-limiting long-chain PUFAs 20:5n-3 and 22:6n-3 decreased under low light acclimation. Also for *C. ovata* pronounced shifts between the proportions of the PUFAs 18:2n-6 and 18:3n-3 and the long-chain PUFAs 20:5n-3 and 22:6n-3 were found: 18:2n-6 and 18:3n-3 decreased, while 20:5n-3 and 22:6n-3 increased, i.e., the shifts were exactly in the opposite direction compared to those observed for *Chromulina* sp.

**Figure 2:**
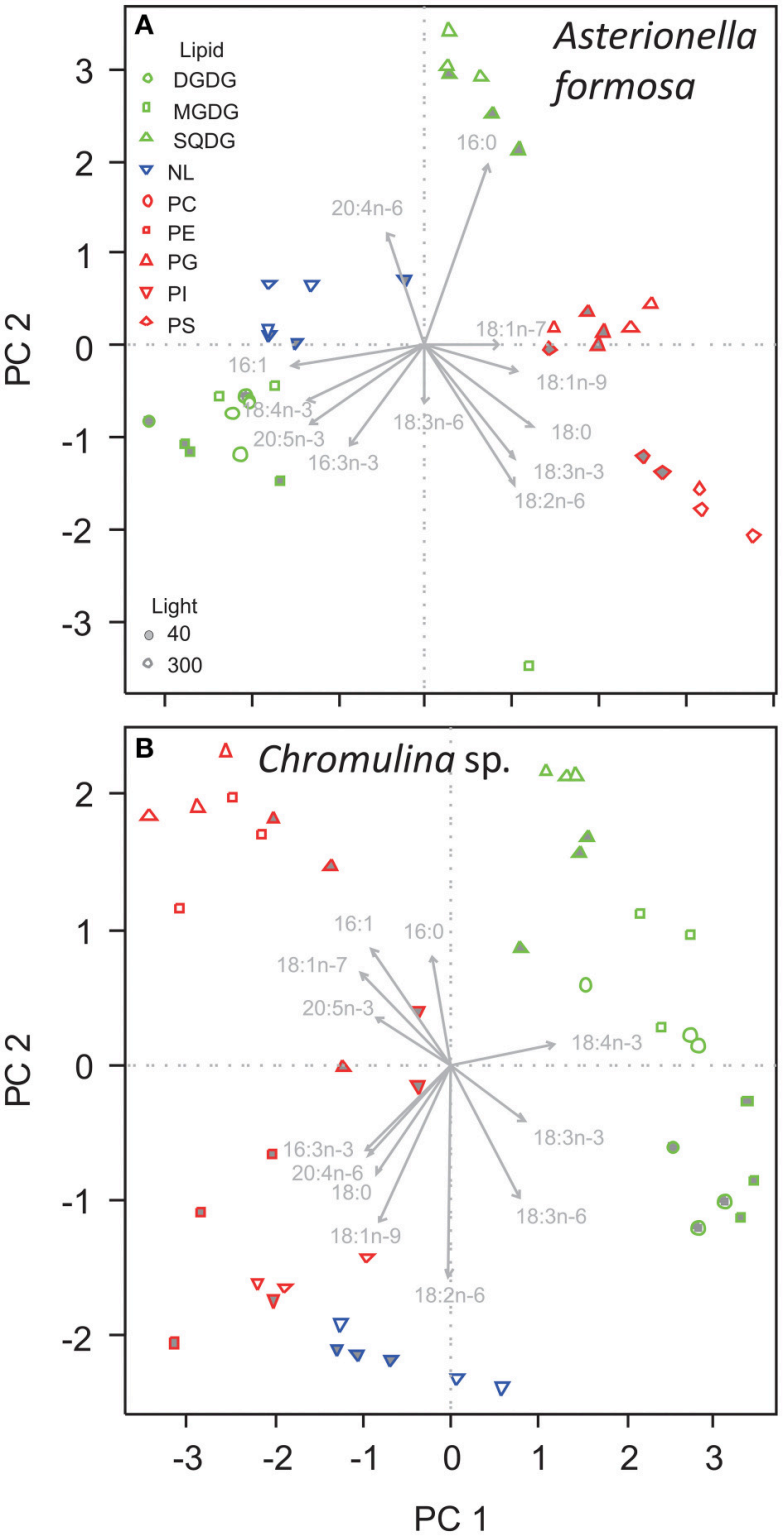
**Principal component analysis of the proportions of fatty acids in different lipid classes of (A) ***Asterionella formosa***, and (B) ***Chromulina*** sp. grown under different light acclimation (40 and 300 μmol photons m^**−2**^ s^**−1**^, filled and open symbols, respectively)**. Glycolipids shown as green symbols are DGDG, digalactosyldiacylglycerol; MGDG, monogalactosyldiacylglycerol; and SQDG, sulfoquinovosyldiacylglycerol. NL, Non-polar lipid are shown as blue triangles. Phospholipids depicted as red symbols are PC, phosphatidylcholine; PE, phosphatidylethanolamine; PG, phosphatidylglycerol; PI, phosphatidylinositol; PS, phosphatidylserine.

The fatty acid composition of *A. formosa, Chromulina* sp., *C. botrytis*, and *C. ovata* were further analyzed by PCA, which projected the maximum variance of the multivariate fatty acid data set on the first principal component axis (PC1); the remaining variance was projected to further orthogonal principal components (i.e., PC1, PC2, etc.). By considering the species separately, we focused closely on the lipid class-specific physiological acclimatization to changes in light. For *A. formosa* PC1 explained 36.3% of variation in the data and clearly separated glycolipids (GL) from phospholipids (PL; Figure 2, Supplementary Table 2). Galactolipids had lower scores on PC1 associated with lower proportions of 18:0 but higher proportions of 16:1n-9 and 20:5n-3 (Table 2). PC2 explained 20.3% and separated SQDG from MGDG, DGDG, and additionally from non-polar lipids (NL). SQDG had clearly higher scores on PC2 than MGDG and DGDG indicating that they contain higher proportions of 16:0 and 20:4n-6 but lower 16:3n-3, and marginal lower 18:2n-6 and 18:3n-3. A comparable trend along PC2 existed for the phospholipids PG and PE. A slight/negligible adjustment of the FA proportions to low light was present along PC2 with 16:0 decreasing in SQDG (Figure 2, Table 2). Adjustments in the galactolipids-associated 16:3n-3 and 18:3n-3 were not found, although a slight tendency of an increase in 20:5n-3 might be present.

**Table 2:** **Fatty acid proportions (%) of the different lipids in ***Asterionella formosa*** grown under different light acclimation (40 and 300 μmol photons m^**−2**^ s^**−1**^)**.

**Lipid Class**	**DGDG**		**MGDG**		**SQDG**		**NL**		**PG**		**PS**	
**Light**	**40**	**300**	**40**	**300**	**40**	**300**	**40**	**300**	**40**	**300**	**40**	**300**
16:0	14.7 (±1.8)	16.3 (±2.0)	14.6 (±0.1)	17.3 (±5.9)	**45.1 (±2.7)**	**50.7 (±0.9)**	25.1 (±7.7)	23.5 (±1.7)	25.9 (±0.7)	28.1 (±2.8)	25.6 (±2.4)	26.4 (±4.6)
16:1n-9	50.9 (±2.3)	48.3 (±3.1)	40.6 (±1.8)	34.4 (±10.8)	21.1 (±3.1)	20.4 (±3.1)	54.8 (±9.7)	56.1 (±1.7)	17.8 (±0.5)	16.3 (±5.3)	18.4 (±5.6)	11.4 (±2.9)
16:3n-3	4.4 (±0.6)	3.2 (±0.8)	10.9 (±0.5)	9.2 (±3.2)	0.8 (±0.3)	0.4 (±0.3)	1.5 (±1.1)	0.7 (±0.2)	1.5 (±0.2)	1.4 (±0.2)	n.d.	n.d.
16:4n-3	n.d.	n.d.	0.5 (±0.1)	0.6 (±0.2)	n.d.	n.d.	0.5 (±0.3)	0.7 (±0.2)	2.3 (±0.5)	1.9 (±0.7)	n.d.	n.d.
18:0	3.5 (±0.8)	4.6 (±1.8)	2.5 (±1.8)	5.9 (±7.2)	6.9 (±4.2)	2.7 (±0.9)	2.5 (±1.8)	2.3 (±0.8)	4.9 (±0.6)	6.3 (±1.7)	**6.9 (±1.7)**	**10.8 (±0.5)**
18:1n-7	1.1 (±1.0)	1.9 (±0.2)	**5.1 (±0.2)**	**4.0 (±0.4)**	3.2 (±0.4)	2.7 (±0.2)	1.2 (±0.4)	1.6 (±0.3)	17.7 (±0.9)	17.4 (±4.2)	3.7 (±0.8)	3.8 (±0.4)
18:1n-9	1.9 (±0.3)	2.6 (±0.5)	2.6 (±0.4)	4.4 (±2.5)	2.1 (±0.7)	1.9 (±0.8)	1.4 (±0.2)	1.2 (±0.1)	16.8 (±2.2)	15.7 (±7.7)	5.2 (±1.6)	6.4 (±0.3)
18:2n-6	1.6 (±0.3)	1.8 (±0.2)	1.5 (±0.2)	3 (±2.5)	1.3 (±0.2)	1.3 (±0.1)	**1.8 (±0)**	**1.2 (±0.2)**	1.9 (±0.2)	1.8 (±0.2)	3.8 (±0.8)	4.5 (±0.2)
18:3n-6	n.d.	n.d.	0.8 (±0.3)	1.5 (±1.1)	n.d.	n.d.	1.1 (±0.2)	1.0 (±0.2)	1.1 (±0.1)	1.2 (±0.3)	n.d.	n.d.
18:3n-3	**1.0 (±0)**	**1.3 (±0.1)**	1.0 (±0.5)	1.0 (±0.3)	0.7 (±0.6)	0.7 (±0.1)	0.6 (±0.1)	0.4 (±0.4)	1.0 (±0.2)	1.1 (±0.1)	2.4 (±0.5)	3.7 (±1.0)
18:4n-3	0.9 (±0.1)	1.5 (±0.7)	0.7 (±0.2)	0.4 (±0.4)	n.d.	n.d.	0.6 (±0.1)	0.7 (±0.1)	n.d.	n.d.	n.d.	n.d.
20:0	1.6 (±0.4)	2.1 (±0.4)	0.6 (±0)	1.0 (±0.4)	1.9 (±0.2)	1.9 (±0.2)	0.9 (±0.7)	0.6 (±0.1)	1.6 (±0.1)	1.9 (±0.5)	4.1 (±0.7)	5.2 (±0.4)
20:3n-6	1.4 (±0.5)	2.5 (±1.8)	0.4 (±0)	0.6 (±0.3)	0.6 (±0.1)	1.1 (±0.7)	1.0 (±1.2)	0.3 (±0.1)	1.3 (±0.3)	1.2 (±0.3)	2.7 (±0.7)	3.9 (±0.8)
20:4n-6	1.8 (±0.3)	1.7 (±0.1)	1.8 (±0.2)	2.3 (±1.1)	3.4 (±0.3)	3.3 (±0.6)	0.8 (±0.2)	1.0 (±0.1)	1.3 (±0.1)	1.4 (±0.3)	n.d.	n.d.
20:5n-3	15.1 (±3.7)	12.2 (±1.6)	15.2 (±1.1)	12.3 (±3.5)	4.2 (±0.5)	4.3 (±0.7)	4.1 (±2.9)	6.3 (±0.8)	5.0 (±0.3)	4.4 (±1.4)	3.2 (±0.4)	3.5 (±0.3)
22:0	n.d.	n.d.	n.d.	n.d.	1.4 (±0)	1.6 (±0.2)	0.5 (±0.2)	0.4 (±0.1)	n.d.	n.d.	4.0 (±0.6)	5.0 (±0.2)
22:6n-3	n.d.	n.d.	0.6 (±0.3)	1.3 (±1.1)	n.d.	n.d.	0.6 (±0.1)	0.5 (±0.1)	n.d.	n.d.	n.d.	n.d.
24:0	n.d.	n.d.	0.5 (±0.1)	0.9 (±0.5)	7.3 (±0.9)	6.9 (±1.5)	1.0 (±0.2)	1.5 (±0.2)	n.d.	n.d.	19.9 (±3.1)	15.5 (±7.8)

*Data shown are means and standard deviations (n = 3). PC, PE, and PI were not detected (n.d.). Numbers in bold/red font indicate statistically significant differences of fatty acid proportions between the two light conditions (ANOVA; p < 0.05). Numbers in bold/blue font indicate statistically significant differences after sequential Bonferroni correction with an effective alpha of 5%. DGDG, digalactosyldiacylglycerol; MGDG, monogalactosyldiacylglycerol; SQDG, sulfoquinovosyldiacylglycerol; NL, non-polar lipid (triacylglycerols); PC, phosphatidylcholine; PE, phosphatidylethanolamine; PG, phosphatidylglycerol; PI, phosphatidylinositol; PS, phosphatidylserine; n.d. = not detected*.

A comparable picture arises for *Chromulina* sp., for which PC1 explained 40.3% of variation in the data and separated GL from PL and NL (Figure 2). GL depicted higher scores on PC1 indicating higher proportions of 18:4n-3 but lower 16:1n-9, 16:3n-3, 18:1n-7, and 20:4n-6 (Figure 2, Supplementary Table 2). PC2 explained 20% of the variation in the data and separated between the different GL. On PC2 SQDG had higher scores than MGDG and DGDG which is associated with lower proportions of 18:2n-6 and 18:3n-6. Here we found that PC2 explained much of the variance in individual PL, e.g., PE changed considerably with light acclimation. In GL the acclimation to low light clearly correlated with PC2. Under low-light acclimation SQDG, MGDG, and DGDG had always lower scores on PC2, which indicates an increase in the proportions of 18:2n-6 and 18:3n-6 in some of the GL (Figure 2, Table 3, Supplementary Table 2). Additionally, low-light acclimation led to a small shift on PC3, which was associated with a two-fold increase of 18:3n-3 in the galactolipid MGDG (Table 3). Under low light proportions of 18:3n-6 were significantly higher in DGDG, MGDG, and SQDG; 18:2n-6 was higher in DGDG, PE, and PG (Table 3).

**Table 3:** **Fatty acid proportions (%) of the different lipids in ***Chromulina*** sp. grown under different light acclimation (40 and 300 μmol photons m^**−2**^ s^**−1**^)**.

**Lipid Class**	**DGDG**		**MGDG**		**SQDG**		**NL**		**PE**		**PG**		**PI/PS**	
**Light**	**40**	**300**	**40**	**300**	**40**	**300**	**40**	**300**	**40**	**300**	**40**	**300**	**40**	**300**
16:0	17.2 (±8.4)	10.7 (±4.5)	5.8 (±1.3)	5.2 (±1.5)	**50.3 (±2.3)**	**44 (±3)**	**15.5 (±0.8)**	**8.6 (±2.7)**	**18.9 (±2.2)**	**24.2 (±2.0)**	17.3 (±4.8)	20.0 (±2.0)	36.7 (±12.8)	26.2 (±2.5)
16:1n-9	1.4 (±0.2)	1.6 (±0.5)	0.8 (±0.3)	0.7 (±0)	1.2 (±0.5)	1.0 (±0.1)	**3.6 (±0.8)**	**2.3 (±0.2)**	**7.7 (±3.1)**	**18.7 (±4.3)**	24.4 (±3.7)	30.3 (±4.9)	**3.9 (±0.9)**	**5.8 (±0.6)**
16:3n-3	0.4 (±0.1)	0.6 (±0.3)	0 (±0.1)	0.2 (±0.2)	0.7 (±0.2)	0.5 (±0.1)	2.4 (±0.1)	1.6 (±0.6)	2 (±0.8)	1.1 (±0.1)	1.0 (±0.2)	1.8 (±1.1)	1.9 (±0.2)	1.7 (±1.5)
16:4n-3	n.d.	n.d.	0.3 (±0.1)	0.4 (±0.2)	n.d.	n.d.	3.4 (±0.2)	4.4 (±2.1)	n.d.	n.d.	n.d.	n.d.	n.d.	n.d.
18:0	3.4 (±2.1)	3.1 (±2.8)	1.2 (±0.2)	1.8 (±1.0)	2.5 (±1.2)	2.5 (±1.0)	7.4 (±0.7)	7.6 (±3.6)	20.7 (±11.4)	9.8 (±5.8)	8.2 (±6.1)	7.1 (±1.1)	16.3 (±18.7)	17.6 (±2.6)
18:1n-7	0.6 (±0.1)	1.0 (±0.4)	0.6 (±0.1)	0.9 (±0.3)	1.0 (±0.6)	0.8 (±0.1)	**3.6 (±0.2)**	**2.2 (±0.2)**	9.4 (±4.5)	17.4 (±3.1)	12.5 (±3.6)	14.4 (±2.1)	3.7 (±1.9)	5.4 (±1.0)
18:1n-9	1.8 (±0.3)	1.5 (±0.5)	1.0 (±0.2)	1.0 (±0.1)	1.7 (±0.6)	1.4 (±0.1)	5.4 (±0.4)	6.8 (±1.1)	**6.6 (±2.0)**	**3.2 (±1.1)**	3.6 (±2.1)	3.2 (±0.7)	7.0 (±5.3)	10.5 (±0.6)
18:2n-6	**5.8 (±0.2)**	**3.6 (±0.8)**	4.7 (±1.1)	2.6 (±1.5)	2.1 (±1.1)	1 (±0.1)	6.8 (±0.3)	6.8 (±1.7)	**4.9 (±1.3)**	**2.7 (±0.5)**	**3.9 (±0.6)**	**2.6 (±0.1)**	**3.2 (±0.4)**	**4.9 (±0.9)**
18:3n-6	**10.1 (±0.8)**	**3.4 (±0.9)**	**13.5 (±2.4)**	**4.2 (±1.0)**	**7.2 (±0.6)**	**1.8 (±0.3)**	8.4 (±0.7)	8.3 (±2.6)	**3.2 (±0.7)**	**1.7 (±0.3)**	2.6 (±1.0)	1.5 (±0.2)	4.8 (±2.1)	3.2 (±0.5)
18:3n-3	21.6 (±5.3)	17.7 (±3.9)	**14.2 (±0.7)**	**7.2 (±0.6)**	2.7 (±1.9)	2.4 (±0)	5.3 (±0.3)	4.8 (±1.3)	4.4 (±1.9)	2.7 (±0.4)	9.3 (±5.9)	3.3 (±0.5)	2.5 (±0.6)	3.7 (±0.7)
18:4n-3	**34.8 (±2.9)**	**52.8 (±5.5)**	**54.4 (±4.1)**	**70.5 (±3.9)**	25.6 (±9.4)	39.0 (±3.4)	**14.1 (±2.4)**	**27.6 (±6.4)**	5.7 (±2.2)	6.3 (±1.6)	8.8 (±9.7)	3.8 (±1.7)	11.6 (±7.7)	7.4 (±1.4)
20:0	1.1 (±0.6)	1.2 (±0.6)	0.6 (±0.3)	0.6 (±0.1)	1.3 (±0.5)	0.9 (±0)	5.7 (±0.5)	4.8 (±0.7)	3.9 (±1.6)	1.8 (±0.2)	1.9 (±0.3)	2.2 (±0.4)	n.d.	n.d.
20:3n-6	0.6 (±0.1)	0.9 (±0.3)	0.5 (±0.2)	0.6 (±0.1)	0.6 (±0.2)	0.5 (±0)	3.0 (±0.4)	2.4 (±0.3)	**2.3 (±0.7)**	**0.4 (±0.7)**	n.d.	n.d.	2.2 (±0.5)	2.0 (±1.9)
20:4n-6	0.5 (±0.1)	0.8 (±0.4)	1.1 (±0.3)	1.5 (±0.6)	0.8 (±0.2)	0.7 (±0.1)	**5.2 (±0.5)**	**3.6 (±0.5)**	7.0 (±2.7)	4.8 (±1.5)	2.7 (±1.2)	2.8 (±0.7)	2.8 (±2.9)	4.2 (±0.6)
20:5n-3	0.7 (±0.1)	1.2 (±0.4)	**0.7 (±0)**	**1.5 (±0.4)**	0.7 (±0.3)	0.7 (±0.1)	3.5 (±0.3)	3.3 (±0.4)	3.4 (±0.7)	4.8 (±2.3)	**3.9 (±0.7)**	**6.9 (±1.1)**	n.d.	n.d.
22:6n-3	n.d.	n.d.	0.3 (±0.3)	0.9 (±0.4)	**1.2 (±0.3)**	**2.5 (±0.7)**	**4.2 (±0.5)**	**3.2 (±0.3)**	n.d.	n.d.	n.d.	n.d.	**3.2 (±0.2)**	**7.3 (±0.7)**

*Data shown are means and standard deviations (n = 3). PI and PS could not be separated and are given as sum; PC was not detected (n.d.). Numbers in bold/red font indicate statistically significant differences of fatty acid proportions between the two light conditions (ANOVA; p < 0.05). Numbers in bold/blue font indicate statistically significant differences after sequential Bonferroni correction with an effective alpha of 5%. DGDG, digalactosyldiacylglycerol; MGDG, monogalactosyldiacylglycerol; SQDG, sulfoquinovosyldiacylglycerol; NL, non-polar lipid (triacylglycerols); PC, phosphatidylcholine; PE, phosphatidylethanolamine; PG, phosphatidylglycerol; PI, phosphatidylinositol; PS, phosphatidylserine*.

**Figure 3:**
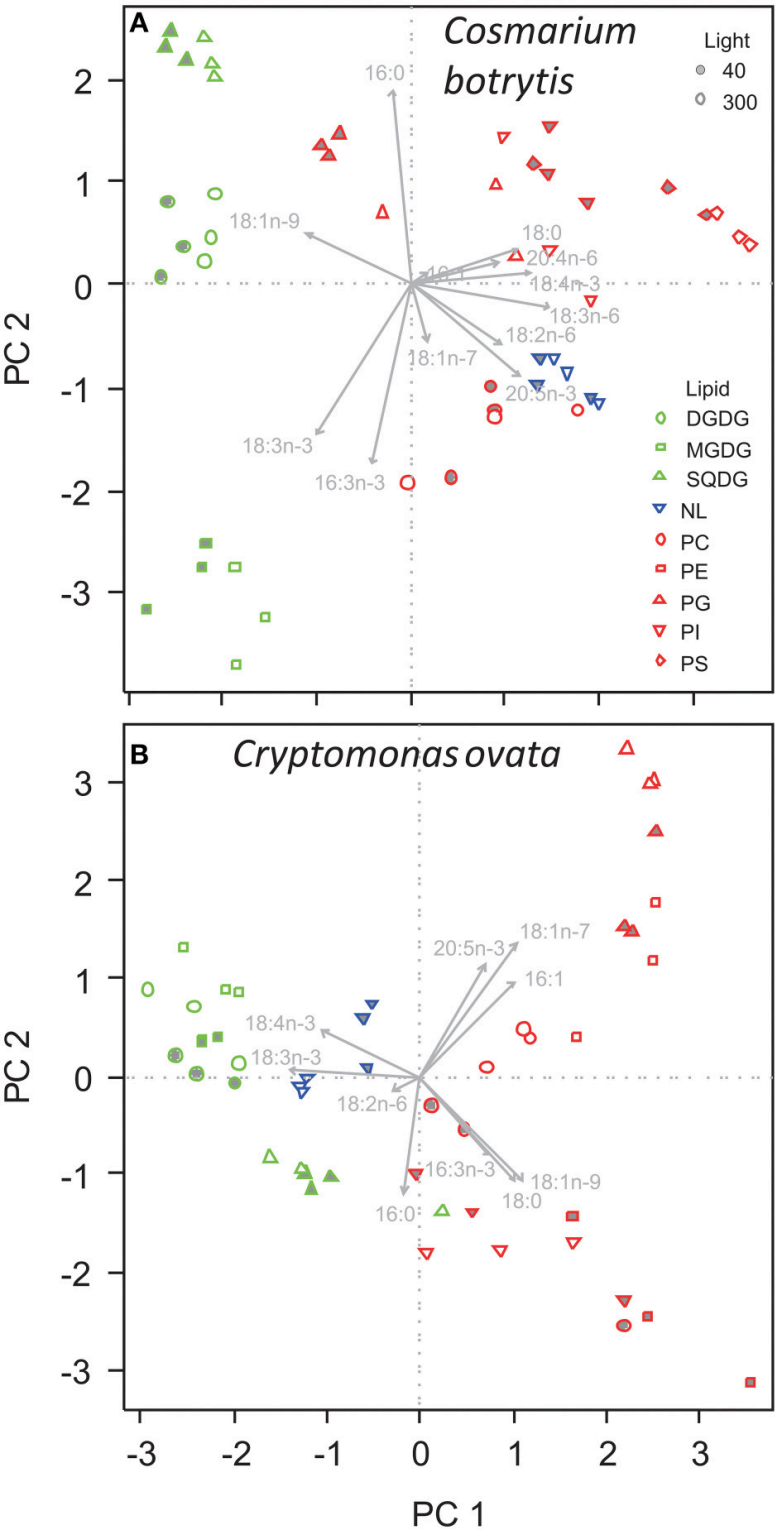
**Principal component analysis of the proportions of fatty acids in different lipid classes of (A) ***Cosmarium botrytis***, and (B) ***Cryptomonas ovata*** grown under different light acclimation (40 and 300 μmol photons m^**−2**^ s^**−1**^)**. Details and color coding of lipids as in Figure 2.

Also for *C. botrytis* GL were separated from PL and NL on PC1, which explained 33.5% of total variation of the data. GL had clearly lower scores on PC1 than PL, which was associated with lower proportions of 18:0, 18:3n-6, 18:4n-3, and 20:5n-3 but (in some GL) higher 18:1n-9. The different PL showed a pronounced variation without a clear segregation along PC1. PC2 explained 21.9% of total variation and separated between the different GL. SQDG had the highest and MGDG the lowest scores on PC2 indicating that SQDG contained high proportions of 16:0 but less of 16:3n-3 and 18:3n-3 whereas the opposite was the case for MGDG (Figure 3, Table 4, Supplementary Table 2). In particular, *C. botrytis* contained high proportions of 16:3n-3 and 18:3n-3 in the two galactolipids MGDG and DGDG (Table 4). The proportions of these fatty acids were generally high, but did not respond to light. 18:3n-3 only slightly increased in PG. Instead, 18:1n-9 increased in all GL and PG (Table 4). In general, the SQDG, MGDG and DGDG as well as PG of low-light acclimated cultures had lower scores on PC1 indicating lower proportions of 18:0, and 18:4n-3 but higher 18:1n-9. Additionally, they showed lower scores on PC3 associated with lower proportions of 16:1. Under low light 18:1n-9 appeared to be generally higher in all lipid classes except PI, and with a trend only in NL and PC (Table 4).

**Table 4:** **Fatty acid proportions (%) of the different lipids in ***Cosmarium botrytis*** grown under different light acclimation (40 and 300 μmol photons m^**−2**^ s^**−1**^)**.

**Lipid Class**	**DGDG**		**MGDG**		**SQDG**		**NL**		**PC**		**PG**		**PI**		**PS**	
**Light**	**40**	**300**	**40**	**300**	**40**	**300**	**40**	**300**	**40**	**300**	**40**	**300**	**40**	**300**	**40**	**300**
16:0	38.8 (±3.3)	45.8 (±3.5)	7.9 (±3.0)	12.9 (±4.1)	59.6 (±1.8)	61.5 (±3.9)	**26.3 (±0.6)**	**31.3 (±1.7)**	23.5 (±3.4)	25.2 (±6.6)	**57.1 (±2.3)**	**34.5 (±5.8)**	49.7 (±7.9)	40.3 (±14.2)	37.6 (±5.4)	36.8 (±1.8)
16:1n-9	**0.6 (±0.1)**	**0.8 (±0.1)**	0.5 (±0.1)	0.8 (±0.2)	0.3 (±0)	0.4 (±0.1)	**1.7 (±0.2)**	**1.0 (±0)**	2.1 (±1.8)	4.5 (±1.7)	**1.6 (±0.4)**	**22.8 (±5.3)**	1.9 (±0.6)	2.6 (±1.6)	n.d.	n.d.
16:3n-3	10.2 (±1.7)	9.6 (±1.5)	30.8 (±3.0)	33.5 (±2.7)	0.3 (±0.1)	1.2 (±1.5)	6.1 (±0.4)	6.0 (±0.5)	18.1 (±4.1)	13.3 (±4.7)	1.7 (±0.3)	3.2 (±3.8)	8.3 (±5.1)	13.7 (±5.1)	**5.1 (±0.8)**	**3.6 (±0.3)**
16:4n-3	n.d.	n.d.	n.d.	n.d.	n.d.	n.d.	1.7 (±0.5)	1.0 (±0.3)	n.d.	n.d.	n.d.	n.d.	n.d.	n.d.	n.d.	n.d.
18:0	1.1 (±0.1)	1.6 (±0.4)	**0.5 (±0.1)**	**0.8 (±0.1)**	1.1 (±0.3)	2.9 (±1.7)	**5.2 (±0.1)**	**3.2 (±0.2)**	10.0 (±3.1)	9.1 (±4.1)	4.9 (±1.9)	7.6 (±4)	13.2 (±6.2)	19.0 (±5.3)	14.8 (±5.4)	10.3 (±0.2)
18:1n-7	0.6 (±0.2)	0.8 (±0)	0.4 (±0.1)	0.6 (±0.1)	0.4 (±0.1)	0.4 (±0.1)	**1.9 (±0.3)**	**1.1 (±0)**	2.2 (±1.9)	5.4 (±2.7)	**1.3 (±0.1)**	**2.5 (±0.3)**	n.d.	n.d.	n.d.	n.d.
18:1n-9	**25.9 (±2.1)**	**18.3 (±0.8)**	**18.6 (±1.4)**	**6.5 (±1.5)**	**27.4 (±2.8)**	**21.5 (±0.7)**	15.7 (±2.1)	12.7 (±1.1)	14.4 (±4.6)	11.2 (±3.6)	**11.4 (±1.2)**	**6.3 (±1.5)**	8.5 (±0.9)	9.0 (±3.6)	**8.9 (±0.9)**	**7.1 (±0.4)**
18:2n-6	2.8 (±0.2)	2.7 (±0.2)	**3.6 (±0.2)**	**2.7 (±0.4)**	1.5 (±0.1)	1.5 (±0)	**15.1 (±0.6)**	**16.1 (±0.2)**	7.6 (±1.2)	5.9 (±0.5)	**3.6 (±0.2)**	**2.4 (±0.3)**	4.6 (±1.1)	3.6 (±0.9)	7.4 (±1.6)	6.7 (±0.5)
18:3n-6	0.4 (±0.1)	0.4 (±0)	0.8 (±0.4)	1.0 (±0.2)	**0.2 (±0)**	**0.3 (±0)**	3.3 (±0.3)	3.6 (±0.2)	3.2 (±0.1)	3.1 (±0.6)	1.9 (±0.2)	1.5 (±0.3)	3.9 (±0.9)	3.4 (±0.8)	3.6 (±3.2)	4.7 (±0.7)
18:3n-3	19.0 (±2.9)	19.0 (±2.6)	34.3 (±2.8)	37.5 (±3.1)	8.9 (±1.6)	9.9 (±1.6)	11.0 (±1.4)	12.7 (±1.1)	12.7 (±8.8)	15.6 (±6.4)	**15.1 (±0.8)**	**12.4 (±1.3)**	2.4 (±0.1)	2.4 (±0.6)	5.4 (±1.2)	5.6 (±1.2)
18:4n-3	0.4 (±0.1)	0.5 (±0.1)	0.9 (±0.4)	1.5 (±0.3)	**0.2 (±0)**	**0.3 (±0)**	**2.7 (±0.2)**	**3.9 (±0.5)**	1.9 (±1.7)	2.8 (±0.3)	1.5 (±0.1)	6.9 (±4.8)	**5.2 (±0.2)**	**2.8 (±0.9)**	5.7 (±1.1)	6.8 (±1.7)
20:4n-6	n.d.	n.d.	0.3 (±0)	0.3 (±0)	n.d.	n.d.	**2.4 (±0.3)**	**1.3 (±0.1)**	n.d.	n.d.	n.d.	n.d.	n.d.	n.d.	9.9 (±4.5)	13.7 (±4.3)
20:5n-3	0.3 (±0.1)	0.4 (±0)	1.4 (±0.7)	1.9 (±0.4)	n.d.	n.d.	5.0 (±0.5)	5.1 (±0.5)	4.2 (±0.4)	4.0 (±0.8)	n.d.	n.d.	2.4 (±0.6)	3.1 (±0.8)	1.6 (±2.7)	4.6 (±0.4)
22:0	n.d.	n.d.	n.d.	n.d.	n.d.	n.d.	**1.9 (±0.4)**	**1.0 (±0.1)**	n.d.	n.d.	n.d.	n.d.	n.d.	n.d.	n.d.	n.d.

*Data shown are means and standard deviations (n = 3). PE was not detected (n.d.). Numbers in bold/red font indicate statistically significant differences of fatty acid proportions between the two light conditions (ANOVA; p < 0.05). Numbers in bold/blue font indicate statistically significant differences after sequential Bonferroni correction with an effective alpha of 5%. DGDG, digalactosyldiacylglycerol; MGDG, monogalactosyldiacylglycerol; SQDG, sulfoquinovosyldiacylglycerol; NL, non-polar lipid (triacylglycerols); PC, phosphatidylcholine; PE, phosphatidylethanolamine; PG, phosphatidylglycerol; PI, phosphatidylinositol; PS, phosphatidylserine*.

For *C. ovata* PC1 explained 35.4% of variation in the data and separated GL from PL (Figure 3). GL had lower scores on PC1 associated with lower proportions of 16:1n-9, 18:0, 18:1n-7, and 18:1n-9 but higher proportions of 18:3n-3 (Table 5, Supplementary Table 2). In addition MGDG and DGDG had higher proportions of 18:4n-3 (Table 5). PC2 explained 21.3% and separated SQDG from MGDG, DGDG, and additionally non-polar lipids. SQDG had lower scores on PC2 than MGDG and DGDG indicating that it contained higher proportions of 16:0 and 18:1n-9 (Table 5). A comparable trend with decreasing scores on PC2 associated with decreasing 18:1n-7 and 20:5n-3 existed for the phospholipids with highest proportions of 18:1n-7 and 20:5n-3 in PG. Under low-light acclimation DGDG and MGDG had higher proportions of 16:0. Additionally, DGDG had higher 18:2n-6 but lower 18:3n-3 (Table 5). In MGDG 18:4n-3 was much lower whereas it increased in SQDG and PG; interestingly, under low light acclimation also 20:5n-3 increased in PG and NL (Table 5). Proportions of 16:3n-3, 18:3n-3, and 20:5n-3 did not increase under low light conditions.

**Table 5:** **Fatty acid proportions (%) of the different lipids in ***Cryptomonas ovata*** grown under different light acclimation (40 and 300 μmol photons m^**−2**^ s^**−1**^)**.

**Lipid Class**	**DGDG**		**MGDG**		**SQDG**		**NL**		**PC**		**PE**		**PG**		**PI**	
**Light**	**40**	**300**	**40**	**300**	**40**	**300**	**40**	**300**	**40**	**300**	**40**	**300**	**40**	**300**	**40**	**300**
16:0	**30.7 (±4.1)**	**18 (±5.3)**	**32.9 (±3.3)**	**14.5 (±4.5)**	59.0 (±6.6)	54.1 (±9.5)	31.3 (±1.1)	29.9 (±0.4)	32.8 (±2.5)	32.6 (±1.1)	29.1 (±5.8)	32.1 (±4.8)	18.8 (±9.6)	17.0 (±2.1)	38.2 (±9.2)	47.9 (±11.8)
16:1n-9	0.9 (±0.1)	1.7 (±0.9)	0.9 (±0.1)	1.6 (±0.6)	0.7 (±0.1)	1.6 (±1.0)	0.9 (±0.2)	1.0 (±0.1)	**3.2 (±0.9)**	**11.5 (±1.7)**	**3.7 (±2.3)**	**22.1 (±2.0)**	**7.8 (±1.4)**	**13.3 (±3.2)**	2.8 (±0.2)	2.9 (±1.9)
16:3n-3	0.7 (±0.1)	0.6 (±0.2)	0.5 (±0)	0.7 (±0.2)	0.7 (±0.1)	1.3 (±1.2)	1.4 (±0.2)	1.1 (±0)	2.3 (±0.4)	1.9 (±0.2)	1.9 (±0.5)	1.2 (±0.2)	1.3 (±0.3)	1.2 (±0.2)	4.6 (±3.5)	2.2 (±1.0)
16:4n-3	n.d.	n.d.	n.d.	n.d.	n.d.	n.d.	1.7 (±0.5)	1.5 (±0.4)	n.d.	n.d.	n.d.	n.d.	n.d.	n.d.	n.d.	n.d.
18:0	3.2 (±2.5)	4.8 (±3.2)	1.8 (±0.7)	3.8 (±3.1)	5.0 (±2.9)	4.3 (±2.7)	4.8 (±0.7)	4.4 (±0.3)	14.5 (±11.7)	7.3 (±1.1)	**30.5 (±10.3)**	**8.8 (±2.4)**	**13.1 (±0.7)**	**4.8 (±1.1)**	10.4 (±3.9)	13.2 (±0.5)
18:1n-7	0.7 (±0.1)	1.3 (±0.9)	**0.8 (±0.1)**	**1.7 (±0.6)**	0.8 (±0.1)	1.5 (±1.9)	**1.0 (±0.2)**	**0.4 (±0.2)**	**3.7 (±0.8)**	**8.7 (±2.4)**	3.9 (±1.1)	14.6 (±7.8)	**17.8 (±2.8)**	**32.0 (±3.6)**	3.2 (±0.2)	2.6 (±1.1)
18:1n-9	1.9 (±0.8)	2.0 (±1.0)	1.0 (±0.3)	1.7 (±0.6)	1.9 (±0.2)	3.0 (±3.0)	**2.7 (±0.8)**	**4.3 (±0.1)**	6.4 (±3)	4.2 (±0.4)	**11.7 (±4.8)**	**5.1 (±0.6)**	**5.0 (±0.2)**	**3.2 (±0.5)**	5.4 (±0.6)	7.2 (±2.9)
18:2n-6	**5.2 (±1.4)**	**2.6 (±0.4)**	4.2 (±1.1)	2.7 (±0.4)	2.1 (±0.4)	3.0 (±1.8)	5.0 (±1.4)	6.6 (±0.4)	4.4 (±1.2)	3.7 (±0.2)	2.3 (±1.6)	2.1 (±0.6)	2.8 (±0.7)	3.3 (±0.3)	4.8 (±0.4)	4.5 (±2.6)
18:3n-3	**35.1 (±1.9)**	**43.0 (±4.4)**	25 (±2.0)	23.2 (±1.7)	22.2 (±1.9)	24.7 (±4.5)	**20.9 (±0.5)**	**28.6 (±0.7)**	9.5 (±3.6)	9.4 (±2.3)	5.6 (±3.8)	6.6 (±2.5)	7.0 (±2.3)	7.5 (±1.3)	14.2 (±6.7)	11.3 (±7.4)
18:4n-3	20.8 (±1.3)	24.9 (±3.6)	**28.2 (±0.8)**	**42.8 (±5.8)**	**3.4 (±0.1)**	**2.2 (±0.7)**	**11.2 (±0.1)**	**13.0 (±0.7)**	8.0 (±3.3)	7.3 (±2.0)	6.6 (±6.3)	2.8 (±1.4)	**5.8 (±0.7)**	**3.0 (±0.9)**	4.5 (±2.1)	2.7 (±1.9)
20:1n-9	n.d.	n.d.	3.1 (±0.4)	5.8 (±1.9)	n.d.	n.d.	1.9 (±0.4)	1.3 (±0.1)	2.4 (±0.9)	2.5 (±0.2)	n.d.	n.d.	1.5 (±1.1)	1.2 (±0.2)	n.d.	n.d.
20:3n-6	n.d.	n.d.	n.d.	n.d.	3.2 (±2.8)	2.6 (±0.5)	n.d.	n.d.	4.0 (±2.6)	3.4 (±1.2)	2.5 (±0.7)	1.1 (±0.9)	n.d.	n.d.	7.9 (±4.4)	2.4 (±1.5)
20:5n-3	1.0 (±0.1)	1.0 (±0.2)	1.7 (±0.2)	1.5 (±0.1)	1.0 (±0.1)	1.5 (±1.0)	**13.7 (±2.5)**	**7.0 (±0.5)**	3.7 (±1.9)	4.1 (±0.6)	2.1 (±1.1)	3.3 (±0.7)	**19.1 (±1.6)**	**13.5 (±1.9)**	4.0 (±0.9)	3.2 (±2.7)
22:6n-3	n.d.	n.d.	n.d.	n.d.	n.d.	n.d.	**3.4 (±0.6)**	**1.0 (±0.1)**	5.3 (±3.8)	3.6 (±0.3)	n.d.	n.d.	n.d.	n.d.	n.d.	n.d.

*Data shown are means and standard deviations (n = 3). PS was not detected (n.d.). Numbers in bold/red font indicate statistically significant differences of fatty acid proportions between the two light conditions (ANOVA; p < 0.05). Numbers in bold/blue font indicate statistically significant differences after sequential Bonferroni correction with an effective alpha of 5%. DGDG, digalactosyldiacylglycerol; MGDG, monogalactosyldiacylglycerol; SQDG, sulfoquinovosyldiacylglycerol; NL, non-polar lipid (triacylglycerols); PC, phosphatidylcholine; PE, phosphatidylethanolamine; PG, phosphatidylglycerol; PI, phosphatidylinositol; PS, phosphatidylserine*.

## Discussion

Our study confirms that phytoplankton species of different taxonomic classes show different FA compositions in their lipid classes. Additional to these species-specific differences the process of acclimating to low light intensities in terms of FA was highly variable among the different species. Our results point out a specific role of the chloroplast lipids (MGDG, DGDG, SQDG, and PG) in the adaptation of algae to low light intensities (Figures 2, 3). Three of the investigated phytoplankton species showed distinct changes of FA proportions with light in MGDG, DGDG, and SQDG. MGDG, DGDG, SQDG, and additionally PG are the four major components of thylakoid membranes (Block et al., 1983) and it has been described for thylakoids of higher plants that each of these lipids has a distinctive role in the photosynthetic membrane (Lee, 2000; Jones, 2007). Interactions of MGDG and the light harvesting complex II (LHC II), for example, are responsible for the stacking of the thylakoid membrane (Lee, 2000; Goss and Wilhelm, 2009; Schaller et al., 2011). MGDG promotes the dimerization of PSII core complexes, and it is indicated that the galactolipid is the major player in the establishment of the native, dimeric PSII structure (Kansy et al., 2014). Furthermore, MGDG and the phospholipid PG are integral parts in the structure of Photosystem I (Jordan et al., 2001), which might be another reason why a particular compositional FA change in photosynthetic membranes at low light took place in MGDG and PG. Under low light we observed ~2–3 fold increases of the n-6 FA 18:2n-6 in PG, 18:3n-6 in MGDG, and both FA in DGDG in *Chromulina* sp. This was the case also for 18:2n-6 in MGDG and PG in *C. botrytis*, and 18:2n-6 in DGDG in *C. ovata*. The opposite alteration took place in the n-3 FA 18:4n-3 and 20:5n-3, which decreased in MGDG and DGDG, and that of 20:5n-3 in PG in *Chromulina* sp. indicating that the molecular composition of chloroplast membranes changed in response to differences in the light environment. As *Chromulina* sp. and *C. ovata* may show phagotrophic behavior, and therefore might be more adapted to low light intensities (Salonen and Jokinen, 1988; Urabe et al., 2000) this may reflect an adjustment process to low light intensity in the thylakoids. In contrast, diatoms such as *A. formosa* are known for their tolerance of large ranges of photon flux densities (Richardson et al., 1983), and therefore might show fewer adjustments of their thylakoid FA composition.

According to studies on chloroplast isolates and galactolipids emphasizing the role of PUFA for maintaining the function of photosystems under light limitation by increasing membrane fluidity and with it electron flow between electron acceptors of Photosystem II (Horváth et al., 1987, Mock and Kroon, 2002, respectively), phytoplankton species should augment their PUFA content under light limiting conditions (e.g., Sukenik et al., 1989; Blanchemain and Grizeau, 1996; Mock and Kroon, 2002).

Important fatty acids associated to thylakoid galactolipids (MGDG, DGDG) are 16:3n-3 and 18:3n-3 (Harwood and Jones, 1989), and were expected to increase under low light conditions. Contrary to expectations, proportions of 16:3n-3 and 18:3n-3 did not increase under low light. An increase of 18:3n-3 was only detected in *Chromulina* sp. In contrast, the observed decrease in n-3 PUFA proportions in some of the species lead to lower fluidity and less activity of the photosynthetic active membranes, which again was the opposite to what was expected. Nevertheless, our partly contradictory observation is consistent with findings of a decreased cellular PUFA proportion at low light (Pronina et al., 1998; Spijkerman and Wacker, 2011), and may result from the fact that at low light conditions a lower fluidity reduces the proton leakage through the thylakoid membrane and energetic maintenance (Raven et al., 2000; Quigg et al., 2006). In general, a separation between the two described opposing compositional responses, (1) lower proportions of PUFA in order to avoid metabolic costs by proton leakage, and (2) higher proportions of PUFA as adaptation to low light conditions (see e.g., Richardson et al., 1983; Neale and Melis, 1986; Sakshaug et al., 1997) appears to be difficult and may depend on species-specific optima and range of light utilization. It might be difficult to extract a general reaction as also other environmental factors such as nutrient provision, preferences in pH and temperature may affect the compositional responses as well.

In order to generalize responses of FA concentrations to environmental conditions we suggest that it might be necessary to know the optimal growth conditions for each alga in terms of light and then to analyze the changes of lipid-specific FA around these optima. In general it is known that green algae and diatoms favor higher light intensities compared to cyanobacteria and flagellates (Wall and Briand, 1979), and diatoms are known to tolerate large ranges of photon flux densities from low to high light (Richardson et al., 1983). The Zygnematophyceae *C. botrytis* shows moderate saturation points for photosynthesis (ca. 200 μmol photons m^−2^ s^−1^ (Maberly, 1983). Cryptophyceae such as *C. ovata* and Chrysophyceae such as *Chromulina* sp. are known to be mixotrophic, and are more adapted to low light intensities (e.g., Salonen and Jokinen, 1988; Urabe et al., 2000). These different species-specific traits and the deviations of our applied experimental conditions from species' optima light intensities might have resulted in apparently different experimental conditions for each species. However, this is the natural situation of phytoplankton species in communities where species coexist because they have different optima and functional traits (e.g., Schwaderer et al., 2011). Consequently, depending on the conditions in experiments and in nature relative to their environmental optimum species may react differently to environmental changes (Richardson et al., 1983; Schwaderer et al., 2011) and adjust their FA composition. The latter might directly influence the photosynthetic adaptation to light intensity (Wacker et al., 2015a). More recently Liu et al. (2016) investigated the fatty acid response of benthic phytoplankton and found it to be dependent on growth phase, nitrogen availability and dark exposure. Nevertheless, their results are clearly in line with our observations that species showed environmentally-induced highly species-specific responses in their FA composition. This supports the fundamental ecological principle of niche differentiation as a mechanism for coexistence among species living in overlapping resource space and time.

In contrast to many other algal species, *Chromulina* sp. and *C. ovata* contained relatively high PUFA proportions in both, GL and NL. Several authors reported that each individual lipid class possessed a characteristic FA pattern (e.g., Sukenik et al., 1993; Khotimchenko and Yakovleva, 2004) and that long-chain PUFA made up a larger proportion in GL than in NL (Guschina and Harwood, 2009), whereas the latter were dominated by saturated fatty acids (SFA) and monounsaturated fatty acids (MUFA). In our study we also observed lipid-class specific FA profiles of GL, PL, and NL with a dominance of PUFA (although not necessarily long-chain PUFA) in GL. Though this is not a common feature, other species have been described that accumulate PUFA in their triacylglycerols, such as the green alga *Parietochloris incisa* (Bigogno et al., 2002) or the red microalga *Porphyridium cruentum* (Cohen et al., 2000).

Our study clearly indicated that light acclimation in algae changed the concentrations of exactly those PUFAs, which *via* altered nutritional quality, may also affect potential consumers at higher trophic levels in natural communities (Hartwich et al., 2012). In particular, shifts between dietary mineral and biochemical components may drive a natural community into co-limitation (Sperfeld et al., 2016), and may hamper efficient energy transfer efficiency across the primary-producer–herbivore interface (Striebel et al., 2012). The present study illustrates the great diversity and variability of lipids and FA in phytoplankton species. In general FA composition of the individual lipid classes was species-specific as well as those FA that were affected by the change in light intensity. Prediction of e.g., phytoplankton food quality changes by using a general model that describes the variation of FA with environmental conditions in phytoplankton communities therefore is still not mature. However, such general models are urgently needed because phytoplankton are the important primary source of PUFA. PUFA play important roles in the cells of all eukaryotic organisms, influence growth and reproduction of herbivorous zooplankton, and thus are important for energy transfer from primary producers to consumers in aquatic food webs (Müller-Navarra et al., 2000; Wacker and Von Elert, 2001; Lukas and Wacker, 2014). Larger organisms, such as fish, cannot synthesize alpha-linolenic acid (18:3n-3) and linoleic acid (18:2n-6), and they can only partly elongate and desaturate 18:3n-3 into eicosapentaenoic acid (20:5n-3) and docosahexaenoic acid (22:6n-3). Therefore, they have to ingest 20:5n-3 and 22:6n-3 as part of their diet (Arts et al., 2001; Parrish, 2009). It appears that aquatic environments are also the main sources of these important FA for terrestrial environments, and consequently, are crucial for human and animal nutrition (Hixson et al., 2015). This emphasizes the need for a comprehensive understanding of environmental changes and their effects on the lipid and FA composition of many more phytoplankton species. For a more comprehensive understanding of acclimatization to light, future studies should also quantify photosynthetic performance (e.g., oxygen evolution, photosynthesis-irradiance curves, photosynthetic capacity, saturation, and compensation points as well as cellular chlorophyll concentrations) in relation to lipid-class specific FA composition.

## Author contributions

AW and MP conceived and designed the study with contribution from MA. MP conducted experiments and analyses. AW, MP, and MA wrote the first draft of the manuscript. IG and JH contributed substantially to interpretation of data and the final version.

### Conflict of interest statement

The authors declare that the research was conducted in the absence of any commercial or financial relationships that could be construed as a potential conflict of interest.

## Abbreviations

ALA: α-linolenic acid
DGDG: digalactosyldiacylglycerol
DHA: docosahexaenoic acid
EPA: eicosapentaenoic acid
FA: fatty acid
FAME: fatty acid methyl ester
GL: glycolipid
LHC: light harvesting complex
LNA: linoleic acid
MGDG: monogalactosyldiacylglycerol
MUFA: monounsaturated fatty acid
NL: non-polar lipid
OD: optical density
PC: phosphatidylcholine
PCA: principal component analysis
PC1: principal component one
PC2: principal component two
PE: phosphatidylethanolamine
PG: phosphatidylglycerol
PI: phosphatidylinositol
PL: phospholipid
POC: particulate organic carbon
PS: phosphatidylserine
PUFA: polyunsaturated fatty acid
SFA: saturated fatty acid
SQDG: sulfoquinovosyldiacylglycerol
TLC: thin-layer chromatography.
