# Supplementary material for: Light-Induced Changes in Fatty Acid Profiles of Specific Lipid Classes in Several Freshwater Phytoplankton Species
Source: Front Plant Sci. 2016 Mar 16;7:264. doi: 10.3389/fpls.2016.00264 (PMC4792871; doi:10.3389/fpls.2016.00264)
Supplement: Supplementary file 1 [file Table1.DOCX]

Supplementary Material

Light-induced changes in fatty acid profiles of specific lipid classes in several freshwater phytoplankton species

**Alexander Wacker*, Maike Piepho, John L. Harwood, Irina A. Guschina, Michael T. Arts**

*** Correspondence:** Alexander Wacker: alexander.wacker@uni-potsdam.de

# Supplementary Figures and Tables

The fatty acid composition of total lipids of *A. formosa*, *Chromulina* sp., *C. botrytis* and *C. ovata* was further analysed by principal component analysis. By considering the total fatty acids of all species together the first three principal components (PC) explained 92% of the variation in the data. PC1 explained 40.3% of the variation and separated *C. botrytis* from the other three phytoplankton species (**Supplementary Figure 1**, **Supplementary Table 1**). *C. botrytis* samples had lower PC1 scores indicating lower proportions of 18:1n-7 but marginal higher proportions of 16:0, 16:3n-3 and 18:1n-9. *Chromulina* sp. was separated from *A. formosa* and *C. ovata* on PC2 which explained 36.4% of the variation in the data. *Chromulina* sp. had lower PC2 scores than *A. formosa* and *C. ovata* associated with lower 20:5n-3 but higher 18:2n-6, 18:3n-6, 18:4n-3 and 20:4n-6. PC3 explained 15.2% of the variation and separated *C. ovata* from *A. formosa* and *Chromulina* sp. as it had lower scores on PC3, which correlated with lower proportions of 16:1n-9 but higher proportions of 18:3n-3 and 18:4n-3. When considering total FA composition of all four species an acclimation to low light was most frequently apparent in *C. botrytis* and *Chromulina* sp. but also to a lesser extent in *C. ovata* (**Table 1**, **Supplementary Figure 1**). In *C. botrytis* the low-light acclimation correlated with PC1 and PC3, associated with higher 18:1n-9 whereas in *Chromulina* sp. low-light acclimation solely appeared along PC2 associated with higher 18:2n-6, 18:3n-6 and 20:4n-6 but lower 20:5n-3.

## Supplementary Figure

**Supplementary Figure 1.** Principal component analysis (PCA) of the proportions of fatty acids in the total lipid fraction of *Asterionella formosa* (Ast), *Chromulina* sp. (Chro), *Cosmarium botrytis* (Cos), and *Cryptomonas ovata* (Cry) under different light acclimation (40 and 300 µmol photons m^-2^ s^-1^, filled and open symbols, respectively). Maximum variance of the multivariate proportions of fatty acids was projected on the first principal component axis (PC1); the remaining variance was projected to the orthogonal principal components two (PC2), etc.

## Supplementary Tables

**Supplementary Table 1.**

Summary of principal component analysis (PCA) of four phytoplankton species using most prominent FA. Maximum variance of the multivariate proportions of fatty acids was projected on the first principal component axis (PC1); the remaining variance was projected to further orthogonal principal components (PC2 and PC3).

|  | total FA of all four phytoplankton species | | |
| --- | --- | --- | --- |
|  | PC 1 | PC 2 | PC 3 |
| Eigenvalue | 4.8 | 4.4 | 1.8 |
| Variance explained (%) | 40.3 | 36.4 | 15.2 |
|  |  |  |  |
| Component loadings | |  |  |
| 16:0 | -0.36 | 0.28 | -0.05 |
| 16:1n-9 | 0.22 | 0.24 | 0.52 |
| 16:3n-3 | -0.42 | -0.03 | 0.22 |
| 18:0 | 0.18 | 0.28 | -0.33 |
| 18:1n-7 | 0.41 | 0.12 | -0.05 |
| 18:1n-9 | -0.40 | 0.02 | 0.22 |
| 18:2n-6 | -0.27 | -0.36 | -0.14 |
| 18:3n-6 | 0.03 | -0.45 | 0.13 |
| 18:3n-3 | -0.35 | 0.05 | -0.46 |
| 18:4n-3 | 0.17 | -0.31 | -0.47 |
| 20:4n-6 | 0.23 | -0.40 | 0.09 |
| 20:5n-3 | 0.06 | 0.42 | -0.21 |

**Supplementary Table 2.**

Summary of principal component analyses (PCA) for each phytoplankton species separately using most prominent FA in different lipid classes. Variance of the multivariate proportions of fatty acids projected on the first four principal component axes (PC1, PC2, PC3, and PC4) is shown.

|  |  | *A. formosa* | |  |  |  | *Chromulina* sp. | |  |  |  | *C. botrytis* | |  |  |  | *C. ovata* | |  |
| --- | --- | --- | --- | --- | --- | --- | --- | --- | --- | --- | --- | --- | --- | --- | --- | --- | --- | --- | --- |
|  | PC 1 | PC 2 | PC 3 | PC4 |  | PC 1 | PC 2 | PC 3 | PC4 |  | PC 1 | PC 2 | PC 3 | PC4 |  | PC 1 | PC 2 | PC 3 | PC4 |
| Eigenvalue | 4.4 | 2.4 | 2.2 | 1.3 |  | 4.8 | 2.4 | 1.9 | 0.9 |  | 4.0 | 2.6 | 1.7 | 1.3 |  | 3.5 | 2.1 | 1.4 | 1.3 |
| Variance explained (%) | 36.3 | 20.3 | 18.0 | 10.8 |  | 40.3 | 19.9 | 16.2 | 7.5 |  | 33.5 | 21.9 | 14.1 | 11.1 |  | 35.4 | 21.3 | 14.0 | 13.0 |
|  |  |  |  |  |  |  |  |  |  |  |  |  |  |  |  |  |  |  |  |
| Component loadings | |  |  |  |  |  |  |  |  |  |  |  |  |  |  |  |  |  |  |
| 16:0 | 0.20 | 0.55 | -0.10 | 0.14 |  | -0.07 | 0.28 | -0.52 | 0.43 |  | -0.06 | 0.58 | -0.05 | -0.09 |  | -0.06 | -0.40 | 0.46 | -0.50 |
| 16:1n-9 | -0.43 | -0.06 | -0.05 | -0.25 |  | -0.31 | 0.29 | 0.34 | -0.05 |  | 0.06 | 0.03 | 0.69 | -0.09 |  | 0.34 | 0.33 | 0.24 | -0.05 |
| 16:3n-3 | -0.24 | -0.31 | 0.27 | 0.45 |  | -0.33 | -0.22 | -0.08 | 0.22 |  | -0.13 | -0.53 | 0.00 | 0.28 |  | 0.25 | -0.27 | -0.38 | -0.21 |
| 18:0 | 0.34 | -0.25 | -0.08 | 0.34 |  | -0.29 | -0.27 | -0.23 | -0.50 |  | 0.35 | 0.11 | 0.12 | 0.24 |  | 0.35 | -0.36 | -0.06 | 0.42 |
| 18:1n-7 | 0.23 | 0.00 | 0.51 | -0.22 |  | -0.35 | 0.23 | 0.28 | 0.02 |  | 0.06 | -0.17 | 0.39 | -0.57 |  | 0.35 | 0.47 | 0.08 | -0.04 |
| 18:1n-9 | 0.29 | -0.08 | 0.43 | -0.21 |  | -0.28 | -0.40 | -0.24 | -0.26 |  | -0.34 | 0.16 | -0.31 | -0.29 |  | 0.38 | -0.36 | -0.18 | 0.25 |
| 18:2n-6 | 0.29 | -0.42 | -0.17 | 0.22 |  | 0.00 | -0.54 | 0.28 | 0.26 |  | 0.29 | -0.18 | -0.29 | -0.42 |  | -0.11 | -0.04 | -0.64 | -0.44 |
| 18:3n-6 | 0.00 | -0.18 | 0.53 | 0.00 |  | 0.27 | -0.34 | 0.13 | 0.46 |  | 0.45 | -0.06 | -0.11 | -0.06 |  |  |  |  |  |
| 18:3n-3 | 0.29 | -0.34 | -0.32 | -0.02 |  | 0.29 | -0.14 | 0.34 | -0.29 |  | -0.30 | -0.44 | 0.03 | 0.14 |  | -0.48 | 0.02 | -0.05 | 0.02 |
| 18:4n-3 | -0.38 | -0.17 | -0.12 | -0.16 |  | 0.41 | 0.05 | 0.06 | -0.17 |  | 0.39 | 0.04 | 0.22 | 0.20 |  | -0.37 | 0.16 | -0.22 | 0.48 |
| 20:4n-6 | -0.12 | 0.34 | 0.17 | 0.63 |  | -0.32 | -0.23 | 0.07 | 0.21 |  | 0.29 | 0.07 | -0.24 | 0.35 |  |  |  |  |  |
| 20:5n-3 | -0.37 | -0.24 | 0.09 | 0.19 |  | -0.29 | 0.12 | 0.44 | 0.08 |  | 0.35 | -0.27 | -0.24 | -0.29 |  | 0.24 | 0.39 | -0.29 | -0.20 |
